# Updates in Anthracycline-Mediated Cardiotoxicity

**DOI:** 10.3389/fphar.2018.01262

**Published:** 2018-11-12

**Authors:** Canan G. Nebigil, Laurent Désaubry

**Affiliations:** CNRS, Laboratory of Biomolecules, UMR 7203, Sorbonne University, Paris, France

**Keywords:** cardio-oncology, cardiotoxicity, chemotheraphy, biomarkers, cardiac damage, anthracylines

## Abstract

Cardiotoxicity is one of the main adverse effects of chemotheraphy, affecting the completion of cancer therapies and the short- and long-term quality of life. Anthracyclines are currently used to treat many cancers, including the various forms of leukemia, lymphoma, melanoma, uterine, breast, and gastric cancers. World Health Organization registered anthracyclines in the list of essential medicines. However, anthracyclines display a major cardiotoxicity that can ultimately culminate in congestive heart failure. Taking into account the growing rate of cancer survivorship, the clinical significance of anthracycline cardiotoxicity is an emerging medical issue. In this review, we focus on the key progenitor cells and cardiac cells (cardiomyocytes, fibroblasts, and vascular cells), focusing on the signaling pathways involved in cellular damage, and the clinical biomarkers in anthracycline-mediated cardiotoxicity.

## Introduction

Either alone or in combination with targeted therapies and cytotoxic agents, anthracyclines are the most commonly used antineoplastic drugs to treat a diversity of hematological and solid tumors (Aleman et al., 2014). For example, doxorubicin (DOX) and its derivative epirubicin are widely used anthracyclines to treat breast, endometrial and gastric cancers, childhood solid tumors, soft tissue sarcomas, and aggressive lymphoblastic or myeloblastic leukemia (Ewer and Ewer, 2015). Daunorubicin is effectively used for treatment of acute lymphoblastic or myeloblastic leukemias, Hodgkin lymphoma and bone sarcoma. Sabarubicin is used for treatment of non-small-cell lung cancer, thyroid and metastatic prostate cancer, and platinum- or taxane-resistant ovarian cancer (Ewer and Ewer, 2015). However, the use of anthracyclines is associated with dose-dependent cardiotoxicity (Table 1). The first year after completing anthracycline chemotherapy, 9% patients had an impaired left ventricular (LV) ejection fraction (EF% < 50%) (Boyd et al., 2017). Delayed HF development is exemplified by long-term childhood cancer survivors having an 12-fold increased chance of developing congestive HF up to 30 years after treatment (Armstrong and Ross, 2014). The occurrence of anthracycline-mediated cardiotoxicity has extended to 30% of adult survivors of childhood cancer (Bhakta et al., 2017). Addition of tyrosine kinase inhibitors with anthracyclines chronic regiments, often implemented for breast cancer treatment, is associated with a 5-year cumulative incidence of heart failure (HF) or cardiomyopathy of 20% (Tsimberidou et al., 2011; Maurea et al., 2016). The DOX cardiotoxicity is manifested as arrhythmias, ischemia, systolic dysfunction and HF, due to cardiac cell death and necrosis (Mitry and Edwards, 2016). Two or three days after administration of DOX, the toxicity among 11% of the patients is manifested as neutropenia, alopecia, nausea, and arrhythmias. A cumulative dose of 550 mg/m^2^ of DOX significantly increases the incidence of development of HF (Swain et al., 2003).

**Table 1:** Comparison of incidence of LV dysfunctions induced by clinically used DOX-derivatives (adapted from Zamorano et al., 2017).

Anthracycline	Incidence of LVSD/HF
DOX (Andriamycin)	7–26% at 550 mg/m^2^
Epirubicin	0.9–11.4% at 900 mg/m^2^
Idarubicin	5–18% at >90 mg/m^2^
MyocetT	2% at 900 mg/m^2^


## Development of New DOX Formulations

**FIGURE 1:**
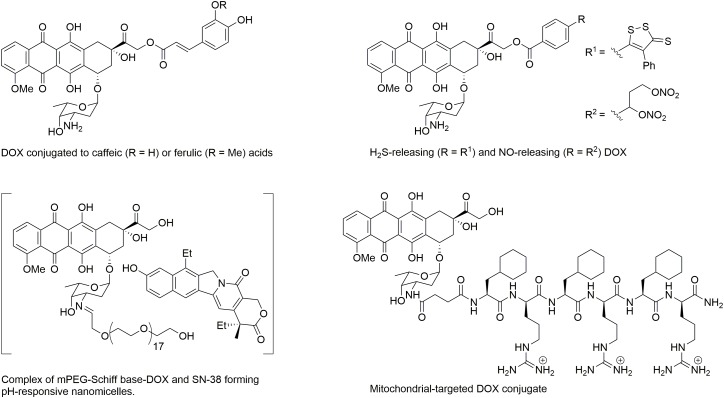
Structure of DOX conjugates displaying a reduced cardiotoxicity.

In recent years, studies have focused on developing novel formulations of DOX to reduce cardiotoxicity without altering its cytotoxic efficacy in cancer cells. A liposomal-delivered DOX has been introduced to prolong circulation levels and control release of DOX (Luo et al., 2017). Pegylated liposomal DOX (PEG-DOX) has found to have a very long circulation time, however, it has high affinity for the skin and induces palmar-plantar erythrodysesthesia in dose dependent manner (Rivankar, 2014). Non-PEG liposomal DOX (MyocetT) has also been developed. MyocetT has a similar cardiotoxicity profile as epirubicin (Table 1), which is lower than the one of DOX (Toldo et al., 2013). However, in the United States, use of liposomal-derived DOX is restricted to ovarian cancer, AIDS-related Kaposi sarcoma, and multiple myeloma (Henriksen, 2018). The other approach to reduce DOX-mediated cardiotoxicity is to use a conjugated co-drug that has cardioprotective effects (Figure 1). For example, conjugating DOX with antioxidant such as caffeic and ferulic acids reduces toxicity as compared to DOX (Chegaev et al., 2013). DOX-conjugated H_2_S (H_2_SDOX) or NO donors (NitDOX) can release thiols and NO, and reduce cardiotoxicity *in vivo*. In the same time, H_2_SDOX and NitDOX reverse chemoresistance in a mouse model of castration-resistant prostate cancer (Chegaev et al., 2016; Bigagli et al., 2018). Another approach is to conjugate DOX to carriers that change the pharmacological distribution of the drug, resulting in reduced drug levels in the heart and targeted delivery of DOX into tumor cells. A pH-responsive nanomicelle composed of mPEG-Schiff base-DOX and 7-ethyl-10-hydroxylcamptothecin (SN-38), an inhibitor of topoisomerase I, not only eradicate breast cancer stem cells, but also enhanced drug accumulation efficiency at the tumor site with lower side effects, and no overt sign of toxicity in heart, liver, spleen, lung, and kidney (Sun et al., 2018). Conjugating DOX with mitochondria penetrating peptide (Chamberlain et al., 2013) generated an adduct called as a mitochondrial-targeted DOX (MtDOX) that can recover cardiomyocytes from mitochondrial damage by activation of compensatory mitochondrial biogenesis without nuclear damage associated with cardiotoxicity (Jean et al., 2015). Although MtDOX is less cytotoxic in drug-sensitive cells, it displays a strong cytotoxic effect in DOX-resistance cells (Battogtokh et al., 2018). Unfortunately, the *in vivo* anticancer activity of these agents has not been reported yet. Isolated mitochondria (Li et al., 2018) or nanomicelles (Zhang et al., 2017) are new nanocarriers that may improve the anticancer efficacy of DOX, but their cardiotoxicity *in vivo* have not been evaluated yet.

The incidence of cardiovascular injuries induced by anthracyclines varies mostly depending on risk factors, genetic predisposition and existence of the cardiovascular disorders, the duration of the therapy and the combinatory cancer therapy. Thus, understanding the mechanism of anthracycline-induced cardiotoxicity, and investigating prognostic value of cardiovascular damage biomarkers in cancer patients could help to avoid and manage it effectively.

## Cellular and Molecular Targets of DOX

**FIGURE 2:**
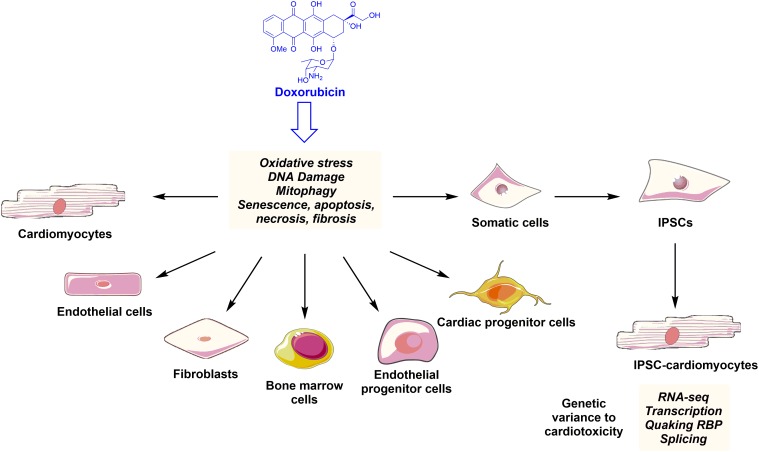
Anthracyclines such as doxorubicin family induce ROS production, DNA damage, apoptosis, and senescence leading to phenotypical and functional changes in the key cardiac and progenitor cells. Indeed, somatic cell derived iPSCs can differentiate into cardiomyocytes. These iPSC-CMs can be used to study new mechanisms of anthracycline-mediated cardiotoxicity and to detect genetic variance to cardiotoxicity in the cancer patients.

Anthracyclines therapies can have a cellular “signature” on the heart that stays latent and asymptomatic at the early stages, ending with a devastating sequel. DOX-mediated structural cardiac damage is associated with changes in several cardiac cell types, leading to cardiac dysfunction and HF (Figure 2). The mechanism of DOX-mediated cardiotoxicity is fairly understood. Mechanisms that have been extensively studied over 5 decades of research, include (1) oxidative stress and generation of reactive oxygen species; (2) topoisomerase II inhibition and double stranded break leading to transcriptional alteration of the genes and apoptosis; and (3) impairments of mitochondrial functions, thereby activating apoptotic pathways. Furthermore the cardiotoxicity is also associated with changes in the high-energy phosphate pool, disturbance of adrenergic signaling and endothelin-1 levels. Here, we will discuss the principle molecular mechanism of DOX-mediated cardiotoxicity in each cardiac cell type.

### Cardiomyocytes

**FIGURE 3:**
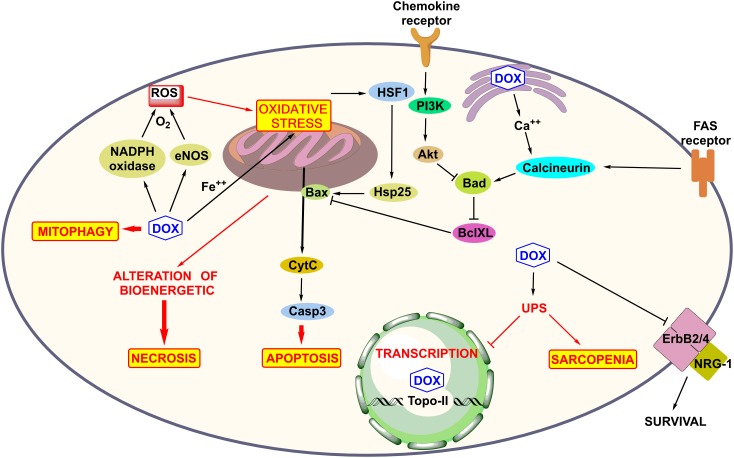
Molecular mechanism of anthracycline-induced cardiotoxicity such as ROS production, mitochondrial and Fas receptor mediated apoptosis, mitophagy, and DNA damage, alteration of bioenergetics, and calcium homeostasis in sarcoplasmic reticulum, activation of UPS system inhibition of NGR-1-mediated survival pathway and role of survival pathway Akt.

Myofibrillar disarray and mitochondrial deterioration in cardiomyocytes are commonly seen structural defects in DOX-mediated cardiotoxicity. The molecular mechanisms of the death of cardiomyocytes have been extensively studied and are underlined below (Figure 3).

#### The Role of Reactive Oxidation Species

Oxidative stress is the most widely studied mechanism for DOX-mediated iatrogenic cardiotoxicity. It results from an imbalance between production of reactive oxygen species (ROS) and reactive nitrogen species (RNS) and intrinsic antioxidant mechanisms that exist at relatively low levels in the heart as compare to other organs. We discuss below how ROS is generated by DOX.

##### Mitochondrial ROS formation and metabolic changes

Mitochondria play a key role as a junction for apoptosis, necrosis, and autophagy processes, and represents one the principal targets of DOX-induced cardiotoxicity. DOX promotes high level of oxidative stress (Angsutararux et al., 2015). When the intra-mitochondrial concentration of DOX surpasses 50–100 μM, the level of ROS production increases. Indeed, a cationic drug DOX attracts cardiolipin, an anionic-charged phospholipid located in the inner mitochondrial membrane. Cardiolipin is implicated in oxidative phosphorylation process and has an important role on the mitochondrial dependent apoptosis. DOX forms an irreversible complex with cardiolipin that is also susceptible to peroxydative attack by ROS (Govender et al., 2014). Cardiolipin peroxidation leads to (i) detachment of cytochrome c from mitochondrial membrane, leading to caspase-depending apoptosis, (ii) uncouples respiratory chain complexes I, III, and IV in the mitochondria, (ii) forms a mitochondrial permeability transition pore (mPTP). Association with Bcl2 family proteins, mPTP leads to reduction of used ATP, thereby inducing necrotic cell death, associated with mitochondrial and cytoplasm swelling, and sarcomere lesions. The necrosis occurs during acute doxorubicin-induced cardiotoxicity. DOX-mediated ROS activates the heat shock factor (HSF)-1, which increases the expression of heat shock protein (Hsp25) and transactivates a tumor-suppressor protein p53, thereby altering levels of beta-cell lymphoma 2–(Bcl2) and pro-apoptotic Bax. Increased Bcl2/Bax ratio triggers the mitochondrial apoptotic pathway in the myocardium (Vedam et al., 2010).

Reactive oxygen species levels are mainly produced by a redox cycling of catalyzed anthracycline in numbers of cellular oxidoreductases (McGowan et al., 2017). This reduction of O_2_^-^ generation is predominantly achieved via nicotinamide adenine dinucleotide (NADH) dehydrogenase (complex I) of the mitochondrial electron transport chain and endothelial-specific nitric oxide synthase (eNOS) reductase that is the most important player in doxorubicin-induced cardiomyopathy among three NOS isoforms (Octavia et al., 2012). DOX depending on the concentration can directly bind to eNOS reductase domain, resulting in O_2_^-^ generation and transforming eNOS from NO to a superoxide producer (Vasquez-Vivar et al., 1997). eNOS-dependent ROS formation contributes to DOX-induced cardiac dysfunction. Interestingly, iNOS also damages DNA by generating peroxynitrites during NO reaction with O_2_^-^, which activates Poly-ADP-ribose polymerase (PARP), leading to an energetic imbalance and cell death, while cardioprotective effect of iNOS is due to generation of NO (Mukhopadhyay et al., 2009).

Doxorubicin-activated PARP unifies the ROS metabolism and DNA repair in cardiomyocytes (Damiani et al., 2016). Although PARP inhibition increases the antioxidant defense and decreases the ROS formation in the H9c2 cells treated with DOX, it is not sufficient to prevent cell death, demonstrating that other molecular signaling contributes to the DOX-mediated cardiotoxicity (Damiani et al., 2018). Use of antioxidants such as vitamin E, vitamin C, carotenoids, flavonoids, polyphenols, etc. together with chemotherapy can be beneficial to minimize the burden of free reactive radicals in cells (Lopes et al., 2017). However, role of antioxidants in cancer therapy are controversial (Yasueda et al., 2016). A preventive low dose of antioxidants has been shown to protect both normal cells and tumor cells, while the therapeutic high dose of antioxidants inhibits the growth of cancer cells, but not normal cells. The optimal doses of antioxidants, and how antioxidant therapy protects normal cells against cardiac damage from cancer therapies, while not affecting their cytotoxic effects in cancer cells need to be determined (Lei et al., 2016).

Doxorubicin also affects mitochondrial metabolism. DOX decreases long-chain-fatty acid oxidation and increases glucose metabolism in mitochondria. DOX controls a switch between an aerobic to anaerobic metabolic state (Carvalho et al., 2010). On the other hand, DOX suppresses cardiac mitochondrial metabolism and biogenesis, and alters metabolic gene expressions. Suppression of mitochondrial metabolism and biogenesis by DOX could be reversed by upregulation of heme oxygenase (HO-1) that is required for mitochondrial biosynthesis system by inhalation of low level of peroxidative carbon monoxide (CO) (Suliman et al., 2007). DOX perturbs the expression of calcium-handling genes, changes the Ca^2+^ homeostasis (Cardoso et al., 2008) and affects fatty acid oxidation (FAO) and metabolism, causing mitochondrial dysfunction and apoptosis in the myocardium.

Doxorubicin-mediated impaired calcium homeostasis can be the result of ROS generation. DOX and its metabolite doxorubicinol induce the calcium/calmodulin-dependent protein kinase-II (CaMKII)-dependent calcium leakage from the sarcoplasmic reticulum, causing calcium overload that leads to sarcomeric disarray, thereby induces necrosis and caspase-12-mediated apoptosis (Sag et al., 2011). DOX down-regulates some of the genes that are involved in the function of cardiomyocytes, including Ca^2+^ ATPase, ryanodine receptor 2 (RyR), mitochondrial iron–sulfur proteins, phospholamban, and calsequestrin (Takemura and Fujiwara, 2007). DOX also activates calpains, calcium-dependent proteases, and induces myofibril deterioration and necrosis (Lim et al., 2004).

##### Iron-dependent reactive oxygen species (ROS) formation

Although oxidative stress has been considered as the central mechanism of DOX-cardiotoxicity, it seems now that iron–DOX complex-induced oxidative stress has a minor role in ROS production (Simunek et al., 2009). DOX undergoes several oxidoreduction processes to form semiquinone metabolites or doxorubicinol, and back to DOX in the presence of iron, leading to O_2_^-^⋅ and H_2_O_2_ formation and apoptosis (Xu et al., 2005).

It seems that DOX-mediated iron accumulation in the cardiomyocytes is more deleterious than the iron-DOX complex-induced ROS production (Ghigo et al., 2016). DOX down-regulates the ATP binding cassette (ABC) B8 protein, a mitochondrial iron export protein, resulting in the diminution of the export of iron from the mitochondria. Cellular iron homeostasis is balanced by the functions of the iron-responsive elements (IREs) and iron regulatory proteins (IRPs). IRPs bind to IREs located in the untranslated regions of mRNAs encoding protein involved in iron uptake, storage, utilization, and export. However, high cellular level of iron promotes the assembly of a [4Fe-4S] cluster that induces the aconitase (ACO) activity of IRPs and abolishes their binding to IRE. DOX or its metabolite DOXol removes Fe^2^ ( + from [4Fe-4S] cluster of ACO1/IRP-1, thereby enhancing stability of mRNA of the iron uptake protein, transferrin, and preventing translation of iron-sequestration protein, ferritin (Canzoneri and Oyelere, 2008)[45]. DOX permanently inactivates both IRP1 and IRP2 (Ichikawa et al., 2014)[46]. Thus, DOX increases the iron integration into the cells and reduces the release of iron from sub-cellular organelles by altering the protein trafficking that promotes iron accumulation inside of the cells.

The hypothesis that mitochondrial oxidative damage caused by iron-induced ROS production has been challenged by the findings that several iron chelator failed to protect from DOX-mediated cardiotoxicity (Simunek et al., 2009; Rao et al., 2011). However, iron chelators such as Dexrazoxane have been used as a preventive therapy. Dexrazoxane has been shown to form an intricate complex with the ATPase domain of human Top2α and Top2β, thereby prevents anthracyclines from binding to Top2, thereby it protects anthracycline-mediated cardiotoxicity (Lyu et al., 2007). An important downside of dexrazoxane is that it has carcinogenic potential with an increased risk for development of acute myeloid leukemia and myelodysplastic syndrome (Shaikh et al., 2016). Therefore, in Europe its use is contraindicated in children, and the European Medicines Agency (EMA) and FDA restrict its use to adult patients with advanced or metastatic breast cancer at high HF risk due to previous receipt of a high cumulative anthracycline dose.

#### The Role of Topoisomerase

Doxorubicin -induced cardiotoxicity does not solely result from the redox cycling of DOX, but also the inhibition of DNA polymerase and nucleic acid synthesis by intercalating with DNA. Indeed, DOX inhibits topoisomerase 2 (Top2) by forming a covalent Top2-DOX-DNA ternary complex (a cleavable complex), leading to double-stranded DNA breaks (Lyu et al., 2007). Top2 is composed of isoenzymes Top2α and Top2β. Top2α is highly expressed during G2/M phases in proliferating (malignant and non-malignant) cells. It is essential for chromosomal segregation (Azarova et al., 2007). Top2α-DOX-DNA complex inhibits DNA replication and chromosome condensation/decondensation, and arrests the cell cycle in G1/G2, thereby inducing apoptosis in proliferating cancer cells. DOX chemotherapy displays a high efficacy, because of the extremely raised expression of Top2α in cancer cells. However, in adult quiescent cardiomyocytes Top2β is particularly abundant, and is constantly expressed (Tewey et al., 1984). DOX exerts cardiotoxicity by intercalating DNA via Top2β in cardiomyocytes. On the other hand, DOX cannot binds to DNA in cardiomyocytes in the absence of Top2β (McGowan et al., 2017). Top2β knockout (KO) mice display a partial-resistance to DOX-induced cardiotoxicity, because of reduced DOX-mediated DNA damage and expression of peroxisome proliferator-activated receptor (PPAR), with a concomitant diminution in p53 induction (Zhang et al., 2012). Importantly, suppression of the PPAR in Top2β-KO mice impairs calcium homeostasis, oxidative metabolism and mitochondrial function, leading to apoptosis. Thus, preventing Top2β degradation can be considered a clinical strategy to protect heart from anthracycline-mediated cardiotoxicity as previously reported (Vejpongsa and Yeh, 2014).

#### The Role of Mitophagy and Autophagy

Mitochondria play an important role for the myocardial contractile function and cell survival, providing sufficient ATP production via oxidative phosphorylation. Defective mitochondria are eliminated by autophagy (mitophagy) that is a fundamental process to sustain mitochondrial network homeostasis, by regulating mitochondrial number and protecting cardiomyocytes from the deleterious effects of ‘mitotoxicity’ (Hamacher-Brady and Brady, 2016). Mitophagy occurs in cardiomyocytes via two pathways: (i) the PTEN-induced kinase 1 (PINK1)/Parkin (E3 ubiquitin ligase) pathway and (ii) BH3-only protein Bcl-2-like 19 kDa-interacting protein 3 (Bnip3)/BNIP3-like protein Nix an effector of apoptosis pathway (Saito and Sadoshima, 2015). Depolarization of outer mitochondrial membrane induces stabilization of PINK1 that binds to Parkin and consequently initiates ubiquitinations and degradation of mitochondrial protein Mitofusin 1 and 2, voltage dependent anion channel-1 (VDAC-1) and GTPase enzyme facilitating mitochondrial transporter, MIRO, thereby preventing mitochondrial re-fusion and promoting mitophagy (Moyzis et al., 2015). Acute DOX exposure promotes Parkin depletion, while post-DOX promotes Parkin upregulation. Indeed, mitophagy inhibitor peptide mdivi-1 prevents the DOX-induced cardiotoxicity, indicating that excessive mitophagy contributes the DOX-cardiotoxicity (Gharanei et al., 2013). Bnip3 or Bnip3L/Nix acts as mitophagy receptor as it has LC3-II recognition motive LIR, thereby allowing autophagosomal engulfment of mitochondria. DOX promotes up-regulation and translocation of Bnip3 and formation of mitochondrial membrane pores, leading to severe necrosis in cardiomyocytes (Dhingra et al., 2014).

Autophagy plays a key role in recycling the cardiomyocyte constituents and is enhanced during cardiomyopathy and HF. However, autophagy can have dual role in cardiomyocytes under stress. DOX treatment causes excessive autophagy, resulting in the degradation of autophagolysosomes in cardiomyocytes via a mechanism that involves iron and ROS to provoke cell death (Nordgren and Wallace, 2014). Strategies aimed at enhancing autophagy before DOX-treatments and preventing post-DOX autophagy initiation have been shown to be cardioprotective (Sishi et al., 2013). For example a basic leucine zipper protein nuclear factor erythroid 2-related factor 2 (Nrf2) prevents oxidative stress by inducing autophagy and protects against DOX-induced cardiomyopathy (Li et al., 2014). Indeed, the silencing of autophagy-related gene (Atg)-5 or the Beclin-1 (Bcl-1) (Xu et al., 2012) or a class III PI3K inhibitor, 3-methyl adenine protect cardiac cells from DOX cardiotoxicity, by inhibiting post-DOX autophagy (Pizarro et al., 2016). More studies will be required to explicate the role of autophagy and mitophagy in DOX-induced cardiotoxicity.

#### Immune Response and the Activation of Death Receptors

Doxorubicin-induced cardiotoxicity can also result from the activation of innate and adaptive immunity. DOX stimulates the release of cytokines and inflammatory markers, such as interleukins (IL) (IL-1β, IL-6) (Sauter et al., 2011), tumor necrosis factor (TNF-α) (Guo R. M.et al., 2013), and mitogen-activated protein kinase (p38 MAPK) and nuclear factor-κB (NFκB) (Guo R. et al., 2013), which are implicated in cardiac pathogenesis and apoptosis. DOX enhances the activity of natural killer cell and cytotoxic T-lymphocytes, and differentiation of the macrophages. DOX also up-regulates the expression of cell surface membrane death receptors (DR), such as Fas cell surface death receptors, DR4, and DR5, TNF receptor 1 (TNFR1) in cardiomyocytes (Zhao and Zhang, 2017). In addition, DOX induces the expression of toll-like receptors (TLR) that play an important role in cardiac damage via activating pro-inflammatory NFκB (Pop-Moldovan et al., 2017). All these DOX-induced immune responses contribute to the activation of a caspase cascade.

#### Neuregulin-1 (NRG-1) (or Erythroblastic Leukemia Viral Oncogene Homolog, ERB)

All four types of NGRs belong to a family of epidermal growth factor proteins. These peptide hormones bind to their tyrosine kinase receptors (ErbBs), and induce dimerization of ERbBs to exert their biological activity. For example NRG1 is a ligand for both ERbB3 and ERbB4, but not for ERbB2. NRG1 binds to ERbB4 and causes its heterodimerization with ERbB2. This ERbB4/ERbB2 signaling activates survival pathway, and induces compensatory hypertrophy in the cardiomyocytes (Lemmens et al., 2007). Chronic exposure of DOX disrupts the expression of NRG1 and ERbB4 in the heart. In accord with this finding, DOX-induced cardiotoxicity was exacerbated in NRG1 knockout mice (Liu et al., 2005). However, acute DOX exposure increases ERbB2 expression, confirming the dose and time dependent anthracycline-mediated cardiotoxicity (Gabrielson et al., 2007). Clinically used ERbB2 antibodies (e.g., Herceptin trastuzumab) for treatment of breast cancer patients directly induce cardiotoxicity, and combination therapy with anthracycline enhances their cardiotoxicity (Nicolazzi et al., 2018). Importantly, engineered bivalent NRGs have been shown to protect mice hearts against DOX-induced cardiotoxicity with reduced neoplastic potential (Jay et al., 2013).

#### Activation of the Ubiquitin Protease System (UPS)

Activation of the UPS system plays a key role in proteolytic degradation and post-translational modification of proteins, and is involved in DOX-mediated cardiomyopathies (Ranek and Wang, 2009). Ubiquitin attachment to the proteins (ubiquitination) requires the ubiquitin-activating enzyme (E1), the ubiquitin conjugating enzyme (E2) and the ubiquitin ligase (E3). DOX up-regulates the expression of E3 ligase and other proteases to promote UPS-mediated degradation of structural proteins (myofibrillar proteins), survival factors (anti-apoptotic Bcl2 protein), transcriptional cofactor p300, nuclear factors of activated T-cells, NFAT-5 and its target gene taurine transporter (TauT) (Lim et al., 2004). The enhanced E3 ligase activity induced by DOX directly promotes myofibrillar loss and apoptosis, and impairs the cardioprotective signaling and the antioxidant amino acid absorption, leading to cardiac dysfunction (Shi et al., 2011).

### Vascular Cells

Recently, DOX-induced toxicity in vascular cells, particularly endothelial injury has received some attention. Blood vessels and coronary endothelial cells are an additional target of anthracyclines (Soultati et al., 2012). DOX alters the nitric oxide/superoxide balance, resulting in the disruption of the endothelial elasticity (Kalivendi et al., 2001). Moreover, DOX lowers NO release and impairs the tube formation and migratory capacity of endothelial cells (Yin et al., 2016). DOX also causes DNA fragmentation-associated apoptosis in endothelial cells (Kaushal et al., 2004). Importantly, vascular endothelial growth factor (VEGF)-B gene therapy reduces DOX-induced apoptosis in endothelial cells and recovers capillary rarefaction, thereby ameliorating the cardiac function in mouse hearts (Rasanen et al., 2016). In accord with this data, VEGF receptor-1 (VEGFR1) has been predominantly found in the cardiac endothelial cells (Kalivendi et al., 2001). Thus, VEGF-B may protect both the endothelial cells and cardiomyocytes (Chen et al., 2010) against anthracycline-induced damage.

Doxorubicin treatment of smooth muscle cells induces pre-mature senescence and severe cellular damage, which is accompanied with ROS production (Bielak-Zmijewska et al., 2014). In addition, DOX-treated vessels display a decrease in alpha-adrenergic receptor levels and exhibit a diminished vessel relaxation, partially because of elevated oxidative stress (Murata et al., 2001). Accordingly, an alpha-adrenergic agonist has a cardioprotective effect in a mouse model of cardiotoxicity that is induced by DOX (Montgomery et al., 2017).

### Cardiac Fibroblasts

Doxorubicin treatment promotes cellular senescence and induces the differentiation of cardiac fibroblasts to a pro-fibrotic phenotype, myofibroblasts (Cappetta et al., 2016). Recently, ataxia telangiectasia mutated (ATM) kinase in cardiac fibroblasts, but not in cardiomyocytes, has been shown to be essential for DOX-induced cardiotoxicity. This finding suggests that fibroblasts might be the principal effector cells of DOX (Zhan et al., 2016). During DOX cardiomyopathy, interstitial fibrosis and perivascular fibrosis have been observed (Carvalho et al., 2014). DOX-mediated cardiac fibrosis occurs as a consequence of necrotic and apoptotic cell damage, and in a pathological response to the excessive ROS production (Zhan et al., 2016). In DOX-induced HF, transforming growth factor-beta (TGFβ) and its downstream-signaling molecules, such as SMAD3, play an important role in stimulating fibrosis (Kuwahara et al., 2002). The contribution of cross talk between cardiac fibroblast and cardiomyocytes in the set of anthracycline cardiotoxicity needs to be further studied.

### Cardiac Progenitor Cells

Doxorubicin-induced long-lasting damage could result from the damage of the quiescent cardiac progenitor cells (CPCs), also called myocardium-resident multi-potent cells (Urbanek et al., 2015). Indeed, DOX has been shown to reduce the viability of c-kit positive CPC *in vivo* that was confirmed by a decrease numbers of CPC in DOX-treated hearts (De Angelis et al., 2010). Interestingly, a cumulative dose of DOX injection shortly after the birth does not induce acute cardiotoxicity in the juvenile mice. However, these mice developed impaired vascular network, fewer CPCs, and had a lower survival rate after myocardial infarction in an adult stage, suggesting that DOX treatment at an early age promotes a higher risk for ischemic injury in the adult heart (Huang et al., 2010). Juvenile DOX exposure also reduces the number of CPCs in heart, indicating that DOX is harmful to these cells (De Angelis et al., 2010). Indeed, DOX changes the telomerase activity in the CPCs by up regulating cell cycle inhibitor p16^INK4a^. Therefore, juvenile exposure, even to a low dose of DOX, induces senescence and permanently reduces the number of resident CPCs (Piegari et al., 2013). Moreover, DOX treatment of human CPCs induces the activation of senescent and pro-apoptotic pathways, indicating that CPC dysfunction leads to a higher susceptibility to myocardial injury. Another recent study has showed that human amniotic fluid stem cell secretoms mitigates DOX-mediated senescence and damage in CPCs (Lazzarini et al., 2016).

In addition to cell death and senescence, DOX alters the function of CPCs by diminishing the growth factor levels, thereby inducing an inadequate response of cardiac repair signaling in the heart. In CPCs, DOX also interferes with the effect of growth factors and hormones, such as the hepatocyte growth factor (Esaki et al., 2008) and testosterone (Ikeda et al., 2010). Moreover, DOX reduces IGF-1R expression in cardiac cells, impairing the cell-protective system and lowering its migratory capacity (Fabbi et al., 2015). DOX-treated CPCs have impaired function in the diseased subject’s myocardium. For example, when DOX-treated human CPCs were administrated to the damaged hearts in mice treated with anthracycline, the hearts did not exhibit any structural and functional recovery, showing the ineffectiveness of DOX-exposed CPCs in the diseased myocardium (De Angelis et al., 2015). Therefore, cell death and senescence, interfered growth factor systems, and the impaired reparative functional properties of CPCs all partially account for DOX-mediated cardiotoxicity (Cappetta et al., 2017).

### Bone Marrow Cells

Bone marrow cells (BMCs) that can differentiate into mesenchymal stem cells (MSCs) are other target cells of DOX (Tomita et al., 2004). Anthracycline induces DNA damage, mitosis, and enzyme inhibition or free radical generation that contribute to the damage of these MSCs. DOX reduces proliferation and differentiation capacity of MSCs in response to cardiomyogenic stimuli (Oliveira et al., 2014) by increasing progressive telomere shortening (Buttiglieri et al., 2011) *in vitro.* Interestingly, anti-cancer drug treatments stimulate apoptosis in MCSs and reduce adipogenic differentiation potential without affecting their chondrogenic differentiation. DOX-mediated ROS production promotes adipogenesis in MCs, but reduces osteogenesis (Atashi et al., 2015). Because MSCs repair DNA breaks to some extent, ROS production by DOX in MSCs could be the main mechanism of cell death.

Granulocyte colony stimulating factor (G-CSF) as a chemokines enhances the migration of BMCs into the heart, and inducing differentiation of BMCs into myocyte-like cells, thereby attenuating cardiotoxicity and improving survival in a mouse model of DOX cardiotoxicity (Tomita et al., 2004). We can assume that DOX damages BMCs-derived stem/progenitor cells in other organs as well (Caplan and Dennis, 2006). The beneficial role of cytokine/chemokine therapy in cardiac regeneration and repair needs to be validated in additional studied in the context of DOX-mediated cardiotoxicity. The long-term effects of anthracyclines on MSCs and bone marrow *in vivo* also need to be investigated.

### Endothelial Progenitor Cells

Physiological or stress-induced stimuli can activate endothelial progenitor cells (EPCs) to regulate angiogenesis and vascular repair (Urbich and Dimmeler, 2004). In an animal model of DOX-induced cardiomyopathy, erythropoietin has been shown to improve myocardial performance by restoring EPC functional properties (Hamed et al., 2006). In EPCs, DOX activates oxidative stress and senescence pathways by regulating p38 and JNK (Spallarossa et al., 2010) or NADPH oxidase (De Falco et al., 2016). Therefore, the induction of senescence and ROS accumulation contributes in the detrimental effects of DOX in EPCs, affecting their function and regenerative capacity. Whether DOX affects the differentiation of EPCs remains to be determined.

### Induced Pluripotent Stem Cells (iPSCs)-Derived Cardiomyocytes (CMs)

A powerful and challenging approach to study genetic predisposition to DOX-mediated cardiotoxicity in human relies on the use of patient-specific iPSC-CMs. Recently, transcriptome analyses in iPSC-CMs revealed that DOX interferes splicing of specific genes that may cause personalized genetic sensitivity to DOX (Knowles et al., 2018). A differential response to DOX has been found between the hiPSC–CMs derived from DOX-treated breast cancer patients without clinical cardiotoxicity (DOX), and DOX-treated breast cancer patients with cardiotoxicity (DOXTOX). More specifically, decrease in viability, perturbations of cellular metabolism and mitochondrial functions, and increases in DNA damage and oxidative stress are higher in the hiPSC-CMs of DOXTOX group than in the hiPSC–CMs of DOX group (Burridge et al., 2016). Using iPSC-CMs, recently, a novel mechanism of DOX-cardiotoxicity has also been described, involving in the alteration (Quaking) of the RNA-binding proteins (RBPs) (Gupta et al., 2018). Briefly, separation of ‘junk’ parts of the RNA from the parts that are used as a template for proteins is disrupted by DOX. Although using iPSC-CM recapitulates *in vivo* inter and intra individual variability in DOX sensitivity, more studies need to be performed on the larger scale.

## Diagnosis/Prognosis of Cardiotoxicity by Clinical Biomarkers

**FIGURE 4:**
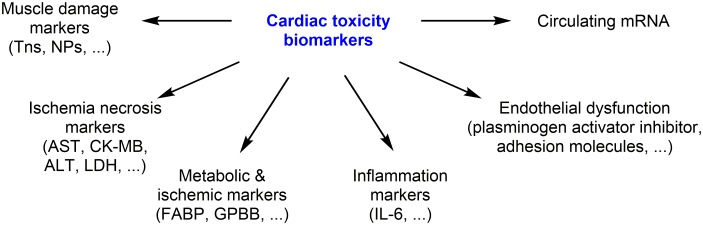
Clinically used biomarkers, and recently identified potential biomarkers.

To assess and monitor the anticancer drug-induced cardiotoxicity cardiac biomarkers has been used for decades. The principal cardiac biomarkers are natriuretic peptides (NPs) and troponins (Tns). Tns are the marker of cardiac injury, while NPs are marker of increased volume expansion and ventricular wall stress. Here, we mainly focus on clinically used biomarkers, and briefly describe recently identified potential biomarkers (Figure 4).

### Troponins

Troponins (Tns) are composed of three subunits: troponin C (cTnC), troponin T (cTnT), and troponin I (cTnI). Troponins make a complex with actinomyosin, and this complex is involved in cardiac contraction and relaxation. In myocardial cells, majority of troponins are located in the sarcomeres and to a lesser extent in the cytoplasm.

Doxorubicin-induced acute cardiotoxicity affects the cell membrane that promotes a rapid depletion of the Tns from the cytoplasmic pool. Chronic exposure to DOX releases detectable Tns into peripheral blood due to the necrosis and rapture of the contractile apparatus (Thygesen et al., 2018). Thus, Tns are considered as cardiac damage markers to evaluate chemotherapy-induced cardiac injury (Cardinale et al., 2017).

A relationship between troponin levels and the degree of late cardiac dysfunction has also been observed in animal treated with the cumulative doses of anthracycline (Wang et al., 2017). In this study the cTnI levels have been found more accurate as compared to cTnT to predict the low-dose anthracycline-induced cardiotoxicity. Persistent increase in cTnT is also positively correlated with anthracycline dose in children treated with anthracycline for lymphoblastic leukemia (Lipshultz et al., 2012). Noteworthy, an increase in troponin cannot be considered a reason to hold or withdraw cancer therapy. However, it can be a tool for identifying patients who have an elevated risk of cardiac dysfunction and need a prophylactic therapy (Cardinale et al., 2017). Recently, high-sensitive (HS) and precise troponin assays have also been applied (Cardinale et al., 2017). However, more comparative research in the larger populations is needed to validate HS-Tn as a prognosis/diagnosis marker of cardiotoxicity.

### Natriuretic Peptides

In response to pressure overload, natriuretic peptides (NPs), including atrial natriuretic peptide (ANP), brain natriuretic peptide (BNP), and BNP’s amino-terminal fragment (NT-pro-BNP), are produced in the atria and ventricles, and released into circulation (Volpe et al., 2016). NPs are implicated in the regulation of vasodilation by inhibiting the sympathetic tone and also in natriuresis and kaliuresis by inhibiting the renin-angiotensin-aldosterone system. Because anthracyclines can induce myocardial ischemia and increase pressure load, a meta-analysis on Asian and Caucasian populations indicated a correlation between elevated BNP levels and cardiotoxicity of anthracyclines (Wang et al., 2016). Although increased NP levels was observed in both pediatric and adult cancer patients who had significant cardiac volume changes by chemotherapy (Christenson et al., 2015), a correlation between an increase in NP levels and the development of cardiac dysfunction has not been confirmed in the large adult population.

### Other Biomarkers

Biomarkers involved in inflammation (high-sensitivity C-reactive protein, interleukin-6), endothelial dysfunction (plasminogen activator inhibitor, soluble intercellular adhesion molecule), myocardial ischemia (fatty acid binding protein, glycogen phosphorylase BB) and NRG-1 have been also considered as diagnostic markers of cardiotoxicity (Curigliano et al., 2016).

Some of the “cardiac enriched” miRNAs (e.g., miR-208, miR-1, and miR-133) or circulating miRNAs have also been implicated in DOX-cardiotoxicity, but these data need to be validated (Ruggeri et al., 2018). Increased levels of markers of myocardial ischemia/necrosis such as serum cardiac enzymes [aspartate aminotransferase (AST), creatinine kinase (CK-MB), lactate dehydrogenase (LDH), and alanine transaminase (ALT)], have also been reported after anthracycline chemotherapy (Cardinale et al., 2017). The correlation between the plasma/serum levels of these biomarkers and clinically defined cardiotoxicity by DOX should be further confirmed and the predictive value of these cardiotoxicity markers should be demonstrated.

## Concluding Remarks

Improvements in cancer therapy and early detection of cancer have increased the survival rate among the cancer patients. However, many anti-cancer treatments, including DOX have cardiac adverse effect, affecting quantity and quality of life. In the future, the development of new DOX formulations targeting only cancer, along with the development of efficient cardioprotectant agents will be of paramount importance in cardio-oncology.

The mechanisms of DOX-mediated cardiotoxicity are multi-factorial and occur because of cell death, such as necrosis, apoptosis, fibrosis, autophagy and mitophagy, and functional changes in cardiac cells independent of injury. Identification of the signaling pathways of anthracycline cardiotoxicity in cardiac cells, dissecting role of cardiac cell communications in the pathophysiology of cardiotoxicity, and interactions with the gender and cardiovascular risk factors such as diabetes and obesity represent an important step toward reducing the risk of morbidity and mortality due to the cardiotoxicity of chemotherapeutics. More importantly a number of genetic variants have been found to predispose patients to the cardiotoxicity of DOX. In future, more studies should be focused on the predictions of a patient’s response to a particular chemotherapy to personalize the cancer treatments, utilizing patient specific hiPSC-CMs. Chemotherapy-induced adverse effect on myocardial contractility through structural and electrophysiological changes can also be studied on hiPSC-CMs (Yang and Papoian, 2018).

Despite, dexrazoxane, angiotensin converting enzyme (ACE) inhibitors, and β-blockade have been proposed as potential preventive strategies, currently there are no clinically proven treatments established for DOX-cardiotoxicity. Recently, Meta analyses have been performed on eight studies (1048 patients), examining the effect of beta-blockers or ACE inhibitors on clinical and sub-clinical cardiotoxicity in patients receiving anthracycline chemotherapy with or without trastuzumab (Gujral et al., 2018). This study has shown that prophylactic ACE inhibitor has no effect on attenuating left ventricular dysfunction or development of HF in these patients. Beta-blocker has a small improvement on left ventricular ejection fraction in patients receiving both therapies, but not in patients receiving anthracycline alone.

Currently, new and advanced cardioprotective drugs are of great interest to protect and cure cardiotoxicity. Several potential cardioprotective drugs that target GPCRs have been identified in the preclinical models of anthracycline cardiotoxicity. For example the antagonists for melatonin (Liu et al., 2002) and cannabinoid CB1 receptor (Mukhopadhyay et al., 2007) protect heart against doxorubicin-induced cardiotoxicity. Some of the tyrosine kinase ligands such as thrombopoietin (Li K. et al., 2006) and erythropoietin (Li L. et al., 2006) also have a cardioprotective role against anthracycline-mediated cardiotoxicity. Moreover, more sensitive biomarkers for prognostic and diagnostic purposes should also be explored to assess whether these markers can be used a diagnostic marker of chemotherapy-induced cardiac damages.

## Author Contributions

All authors listed have made a substantial, direct and intellectual contribution to the work, and approved it for publication.

## Conflict of Interest Statement

The authors declare that the research was conducted in the absence of any commercial or financial relationships that could be construed as a potential conflict of interest.
